# DREAM (Downstream Regulatory Element Antagonist Modulator) contributes to synaptic depression and contextual fear memory

**DOI:** 10.1186/1756-6606-3-3

**Published:** 2010-01-21

**Authors:** Long-Jun Wu, Britt Mellström, Hansen Wang, Ming Ren, Sofia Domingo, Susan S Kim, Xiang-Yao Li, Tao Chen, Jose R Naranjo, Min Zhuo

**Affiliations:** 1 Department of Physiology, Faculty of Medicine, University of Toronto, 1 King's College Circle, Toronto, Ontario, Canada; 2 Department of Molecular and Cellular Biology, National Centre of Biotechnology, Consejo Superior de Investigaciones Científicas, E-28049 Madrid, Spain; 3 Department of Brain and Cognitive Sciences, Seoul National University, Seoul 151-746, Korea

## Abstract

The downstream regulatory element antagonist modulator (DREAM), a multifunctional Ca^2+^-binding protein, binds specifically to DNA and several nucleoproteins regulating gene expression and with proteins outside the nucleus to regulate membrane excitability or calcium homeostasis. DREAM is highly expressed in the central nervous system including the hippocampus and cortex; however, the roles of DREAM in hippocampal synaptic transmission and plasticity have not been investigated. Taking advantage of transgenic mice overexpressing a Ca^2+^-insensitive DREAM mutant (TgDREAM), we used integrative methods including electrophysiology, biochemistry, immunostaining, and behavior tests to study the function of DREAM in synaptic transmission, long-term plasticity and fear memory in hippocampal CA1 region. We found that NMDA receptor but not AMPA receptor-mediated current was decreased in TgDREAM mice. Moreover, synaptic plasticity, such as long-term depression (LTD) but not long-term potentiation (LTP), was impaired in TgDREAM mice. Biochemical experiments found that DREAM interacts with PSD-95 and may inhibit NMDA receptor function through this interaction. Contextual fear memory was significantly impaired in TgDREAM mice. By contrast, sensory responses to noxious stimuli were not affected. Our results demonstrate that DREAM plays a novel role in postsynaptic modulation of the NMDA receptor, and contributes to synaptic plasticity and behavioral memory.

## Introduction

Long-term plastic changes in the individual synapses of the central nervous system are engaged in key physiological functions such as neurodevelopment, learning, memory, fear and emotion, and in many pathological conditions such as drug abuse, chronic pain, anxiety and other brain diseases [1-7]. Calcium-dependent signaling pathways, triggered mainly by several neurotransmitter receptors and voltage-dependent ion channels, contribute to gene regulation and protein synthesis that are critical for long-term plastic changes [2,8-10]. The signaling pathways linking calcium increases to gene activation have been intensively investigated in synaptic plasticity and behavioral memory. For example, cAMP, produced by calcium-calmodulin (CaM) activated adenylyl cyclases (AC) including AC1 and AC8, regulates new gene expression and protein synthesis through PKA-CREB dependent pathways, and directly or indirectly contributes to long-term potentiation (LTP) [2,11,12] and memory from invertebrates to vertebrates [13-18]. Furthermore, calcium-CaM regulated protein kinases including CaMKII and CaMKIV are also critical in synaptic plasticity and behavioral memory [19-27]. Both PKA and CaMKIV regulate the activation of CREB in the nucleus either through the subunit translocation (in case of PKA) or the entry of CaM (CaMKIV) [28-30].

In addition to well-known protein kinases and phosphatase pathways, postsynaptic calcium also regulates the transcriptional activity of some repressors [8,31-33]. The DREAM was characterized as a multifunctional Ca^2+ ^binding protein with defined functions both in and outside the nucleus. In the nucleus, DREAM binds to specific downstream regulatory element (DRE) to repress transcription of target genes [34-36]. Outside the nucleus, DREAM interacts with presenilins or Kv4 potassium channels, to modulate calcium release from the endoplasmic reticulum [37] or channel gating, respectively [38,39]. Because of that, DREAM is also known as calsenilin or potassium channel interacting protein-3 (KchIP-3) [39,40]. The roles of DREAM in synaptic plasticity and behavioral learning have been investigated and the potential phenotype in learning-related plastic pathways observed in DREAM knock out mice [35,41,42]. Due to genetic compensation by the other members of the DREAM/KChIP family, the use of DREAM/calsenilin knockout mice in the study of synaptic transmission, synaptic plasticity and memory could have compromised the observation of a clear phenotype [32,33].

In the present study, we used transgenic mice (TgDREAM) overexpressing a Ca^2+^- and cAMP-insensitive DREAM mutant, which has been shown to act as a dominant active mutant specific for DREAM transcriptional repressor function, blocking basal expression of Na^+^/Ca^2+ ^exchanger 3 (NCX-3) in the brain [43] and cytokines in transgenic T cells [44]. By integrating different approaches, including electrophysiology, biochemistry and pharmacology, the roles of DREAM in basal synaptic transmission and long-term plasticity in hippocampal CA1 neurons were investigated. We found that NMDA receptor-mediated synaptic transmission and plasticity was impaired in TgDREAM mice. Co-immunoprecipitation results indicated that DREAM interacts with PSD-95. Consistently with synaptic findings, the TgDREAM mice showed impairments in contextual fear memory. Our results provide strong evidence that DREAM modulates the function of postsynaptic NMDA receptor, synaptic plasticity, and behavioral learning and memory.

## Results

**Figure 1:**
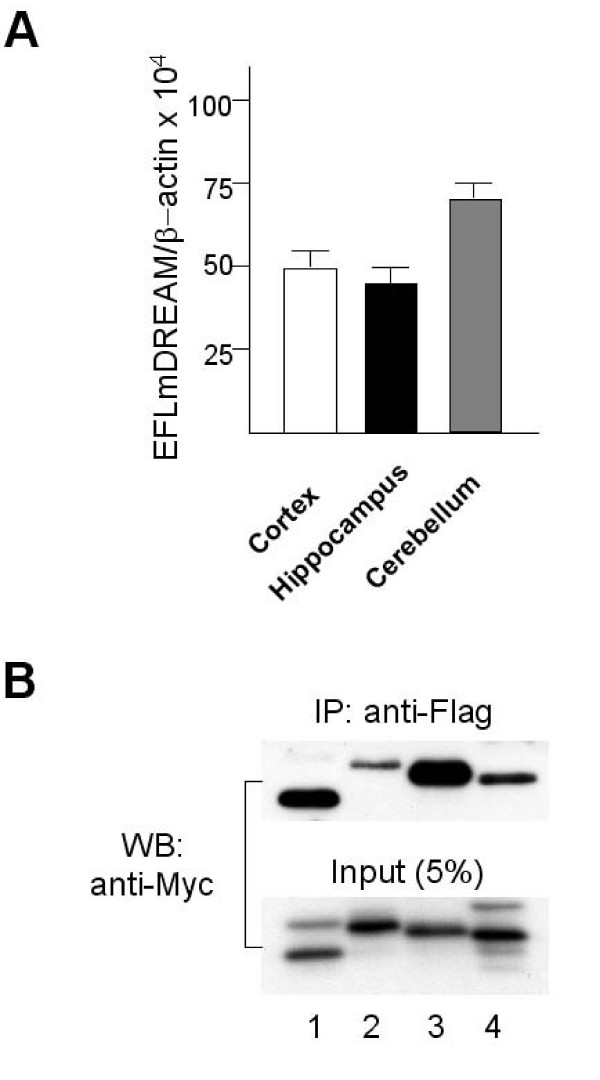
**In vivo and in vitro characterization of TgDREAM**. **(A) **Quantitative real-time RT-PCR of TgDREAM mRNA levels in different brain areas from transgenic mice. Expression level of the transgene was corrected by β-actin mRNA level in each ample. **(B) **Co-immunoprecipitation of TgDREAM-Flag and DREAM/KChIP Myc-tagged proteins (1 to 4) after overexpression in HEK293 cells.

**Figure 2:**
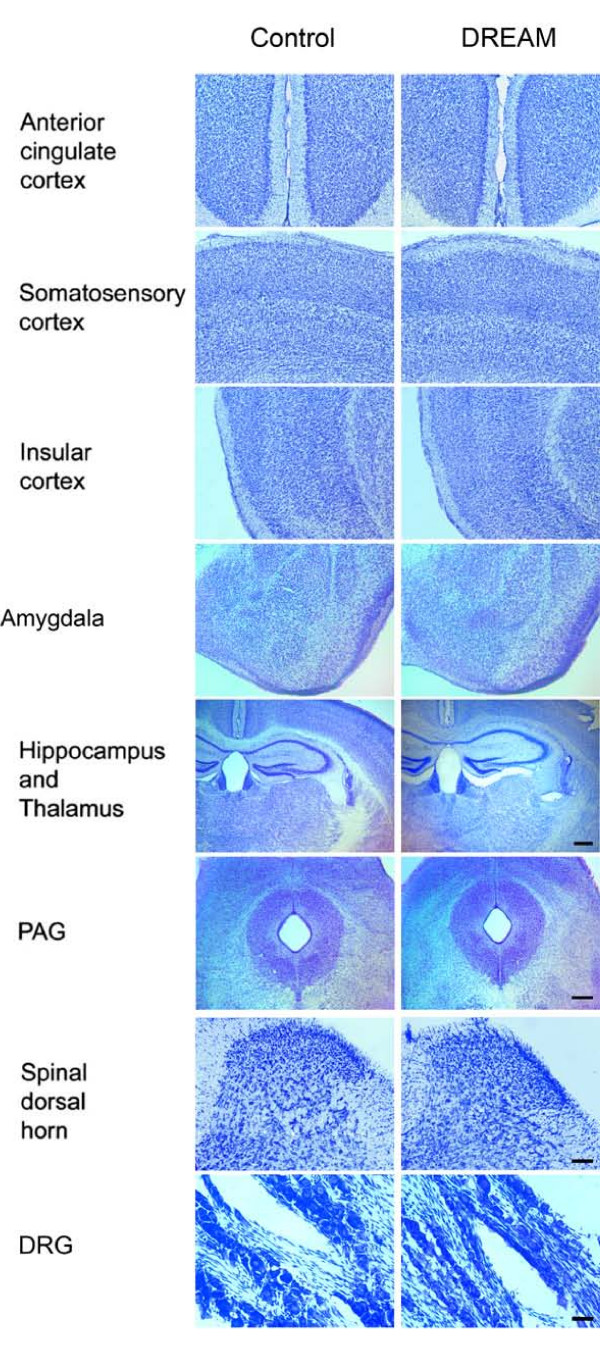
**Normal brain morphology in TgDREAM mice**. Coronal sections showed no detectable morphological differences in the anterior cingulate cortex, somatosensory cortex, insular cortex, amygdala, hippocampus, thalamus, periaqueductal gray (PAG), spinal cord dorsal horn as well as dorsal root ganglia. Scale bar: 250 μm (cortex, amygdala and PAG), 500 μm (hippocampus and thalamus), 100 μm (spinal dorsal horn) and 50 μm (dorsal root ganglia).

To analyze the functional significance of the Ca^2+^-dependent transcriptional repressor DREAM proteins, we used transgenic mice that express the double DREAM mutant that will block specifically Ca^2+ ^dependent derepression at DRE sites [43] without affecting CREB-dependent transcription [45]. Quantitative RT-PCR showed that mutant DREAM is expressed in the hippocampus, cerebral cortex and cerebellum (Figure 1A). We then analyzed whether mutant DREAM also interacts physically and forms heterooligomers with KChIP-1 and -4 proteins. Co-immunoprecipitation of differentially tagged Ca^2+ ^insensitive mutant DREAM and DREAM/KChIP proteins (1, 2, 3 and 4) confirmed the cross-interaction between DREAM and the other three family members after overexpression in HEK293 cells (Figure 1B). Thus, in the brain the mutant DREAM is likely interfere with all Ca^2+^-related transcriptional responses mediated by DREAM/KChIP proteins. We also studied the gross anatomy of the brain in transgenic mice by Cresyl violet staining. Serial coronal sections were examined and there is no detectable morphological difference between TgDREAM and wild-type mice in the anterior cingulate cortex, somatosensory cortex, insular cortex, amygdale, hippocampus, thalamus, periaqueductal gray, spinal dorsal horn, or dorsal root ganglia (Figure 2).

### Loss of LTD in TgDREAM mice

**Figure 3:**
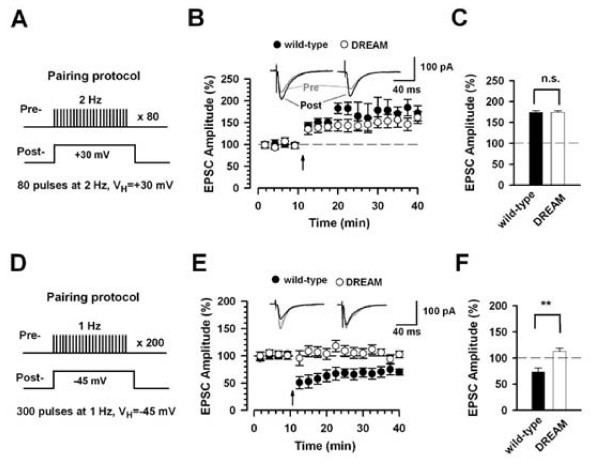
**Reduction of pairing protocol-induced long-term depression in the hippocampus in TgDREAM mice**. **(A) **Diagram showing the paring protocol for LTP induction. The holding potential is +30 mV and 80 presynaptic stimulations at 2 Hz were applied. **(B) **LTP induced in hippocampal CA1 pyramidal neurons in wild-type mice (filled circles, n = 6) and TgDREAM mice (open circles, n = 6) by pairing protocol. In the present and following figures, the insets show averaged EPSC at 5 and 25 min after the pairing procedure (arrow). The dashed line indicates the mean basal synaptic response. **(C) **Pooled data show no significant difference (n.s.) of LTP in wild-type and TgDREAM mice. **(D) **Diagram showing the pairing protocol for LTD induction. The holding potential is -45 mV and 300 presynaptic stimulations at 1 Hz were applied. **(E) **LTD was stably induced by pairing protocol in wild-type mice (filled circles, n = 8), but completely abolished in TgDREAM mice (open circles, n = 8). **(F) **Pooled data show a significant difference in LTD between wild-type and TgDREAM mice. ** *P *< 0.01.

As a multifunctional Ca^2+^-binding protein highly expressed in neurons, DREAM is an attractive candidate serving critical roles in brain functions. Few studies, however, have aimed to address this question, particularly hippocampus-related functions [35,41]. Here we focused on the functions of DREAM in the synaptic transmission and plasticity, as well as hippocampus-related memory. First, we examined the hippocampal Schaffer collateral pathway in the CA1 region and compared electrophysiological responses in wild-type versus TgDREAM mice. Experiments were performed by conventional whole-cell patch clamp recordings in visually identified pyramidal neurons from hippocampal CA1 layer. Evoked excitatory postsynaptic currents (EPSCs) were obtained by delivering stimuli using a bipolar electrode placed in the stratum radiatum. To induce LTP in hippocampal CA1 neurons, the pairing protocol involving holding CA1 neurons at +30 mV paired with 80 pulses of presynaptic stimulation at 2 Hz was used (Figure 3A). LTP was reliably induced in wild-type mice (173.1 ± 1.5% of baseline response, n = 6) (Figure 3B and 3C). Similar LTP was observed in TgDREAM mice (174.6 ± 1.5% of baseline response, n = 6) (Figure 3B and 3C), indicating that LTP is not affected in transgenic mice.

LTD was then tested in TgDREAM mice. For that, low frequency synaptic stimuli (1 Hz) paired with CA1 neurons held at -45 mV were used (Figure 3D). LTD was triggered in wild-type mice (73.0 ± 4.6% of baseline response, n = 8) (Figure 3E and 3F). In contrast, LTD was significantly reduced in TgDREAM mice (112.5 ± 6.8% of baseline response, n = 8, *P *< 0.01 compared with LTD in wild-type mice) (Figure 3E and 3F). Taken together, these results indicate that DREAM contributes selectively to pairing protocol-induced LTD but not LTP induction in the hippocampal CA1 pyramidal neurons.

### Reduced NMDA receptor-mediated EPSCs in TgDREAM mice

**Figure 4:**
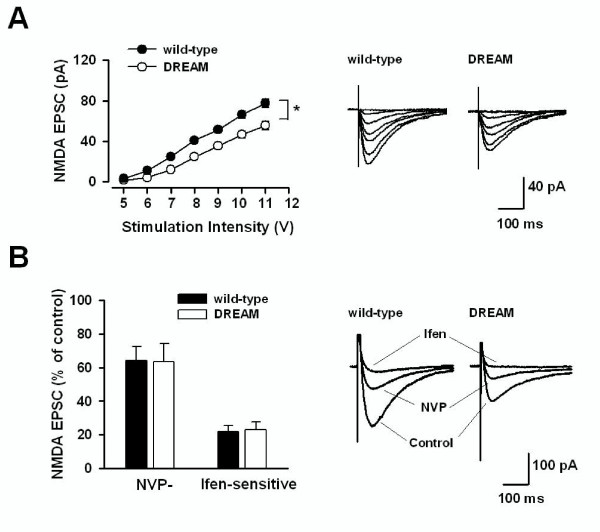
**Reduced NMDA receptor-mediated synaptic transmission in TgDREAM mice**. **(A) **Input-output relationship for NMDA receptor-mediated EPSCs evoked by various stimulation intensities in wild-type mice (filled circles, n = 8) and TgDREAM mice (open circles, n = 7). The amplitude of NMDA receptor current in TgDREAM mice was significantly reduced compared with that in wild-type mice. * *P *< 0.05. The right panel shows representative traces for NMDA receptor-mediated EPSCs in wild-type and TgDREAM mice. **(B) **The percentage of NR2A or NR2B component of NMDA receptor-mediated EPSCs is similar in the TgDREAM (n = 7) and wild-type mice (n = 7). The right panel shows sample traces of NMDAR-mediated EPSCs in control, 0.4 μM NVP-AAM077 (NVP) and 0.4 μM NVP-AAM077 + 3 μM ifenprodil (NVP + Ifen) in wild-type and TgDREAM mice.

Pairing protocol-induced LTD in the hippocampal CA1 synapse required postsynaptic Ca^2+ ^elevation through activation of the NMDA subtype of glutamate receptors. Therefore, we tested the function of NMDA receptor complex in TgDREAM mice. Experiments were performed to compare NMDA receptor-mediated EPSCs in TgDREAM and wild-type mice. Various stimulation intensities were used and input-output relationship of NMDA receptor-mediated EPSCs were generated. We found that the amplitude of NMDA receptor-mediated EPSCs in TgDREAM mice (n = 7) was significantly reduced compared with that in wild-type mice (n = 8, *P *< 0.05) (Figure 4A).

Functional NMDA receptors in the hippocampal CA1 neurons contain mainly heterogenic combinations of the NR1 with NR2A or NR2B subunits [46]. Next we wanted to know the contribution of NR2A and NR2B to the reduction of NMDA receptor-mediated currents in TgDREAM mice. For that, pharmacological antagonists for NR2A or NR2B were used. Bath application of NVP-AAM077 (0.4 μM), a relatively selective NR2A subunit antagonist, blocked NMDA receptor-mediated EPSCs by 64.4 ± 8.3% (n = 7) in wild-type mice and by 63.2 ± 10.7% (n = 7) in TgDREAM mice (Figure 4B). Following the addition of ifenprodil (3 μM), a selective antagonist for NR2B subunit, EPSCs were further decreased by 21.6 ± 3.7% (n = 7) in wild-type and by 22.5 ± 4.8% (n = 7) in TgDREAM mice (Figure 4B). These results indicate that both NR2A and NR2B-containing NMDA receptors were impaired in transgenic CA1 hippocampal neurons.

### Normal expression and phosphorylation of NMDA receptor subunits in TgDREAM mice

**Figure 5:**
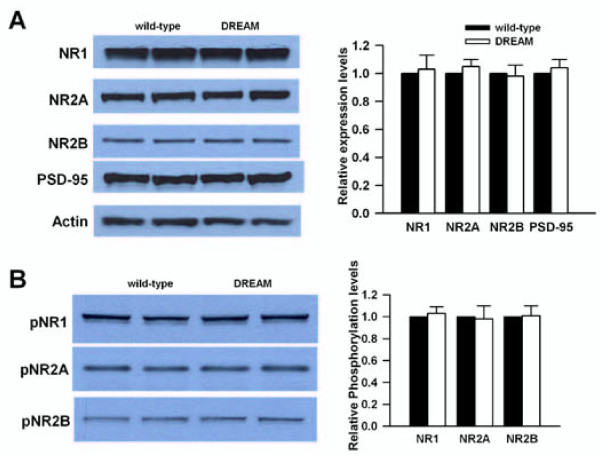
**Analysis of NMDA receptor protein in the hippocampus of TgDREAM mice**. **(A) **Representative western blot (left) and quantified data (right) for expression levels of NR1, NR2A and NR2B subunits, and PSD-95 in hippocampus from wild-type and TgDREAM mice. Data were normalized to expression level of wild-type mice (n = 4 for each group). **(B) **Representative western blot (left) and quantified data (right) for phosphorylation of NR1, NR2A and NR2B at serine residues in hippocampus from wild-type and TgDREAM mice. Data were normalized to expression level of wild-type mice (n = 4 for each group).

The reduced NMDA EPSCs may be due to the altered expression or phosphorylation of NMDA receptor subunits. Using Western blot, we found no difference in the expression of NMDA receptor protein subunits, NR1, NR2A or NR2B, in wild-type and TgDREAM mice (Figure 5A), suggesting that the genes encoding these subunits are not targets for DREAM repression. Moreover, we found that the levels of PSD-95 protein, a well-characterized NMDA receptor interacting protein, were similar in TgDREAM and wild-type mice (Figure 5A).

Phosphorylation of NMDA receptors is important for their synaptic functions [47,48]. We next wanted to know whether the phosphorylation of NMDA receptors was modified in TgDREAM hippocampus. To address this question, we studied total phosphorylation of NMDA receptor subunits in wild-type and TgDREAM mice. We found no change in the phosphorylation of NMDA receptor subunits NR1, NR2A or NR2B in TgDREAM mice (Figure 5B). Taken together, our results suggest that DREAM is not directly involved in the regulation of NMDA receptor expression or phosphorylation.

### DREAM interacts with PSD-95

**Figure 6:**
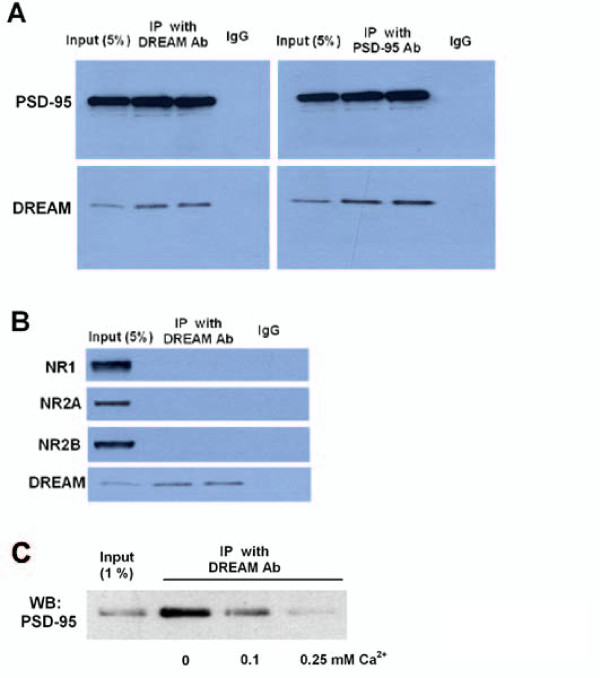
**DREAM interacts with PSD95 protein in vivo**. **(A) **Co-immunoprecipitation of DREAM with PSD-95 in mouse hippocampal extracts from wild type mice. DREAM antibody immunoprecipitated DREAM bound to PSD-95 (left) while the PSD-95 antibody immunoprecipitated PSD-95 along with DREAM (right). Neither PSD-95 nor DREAM was immunoprecipitated using a control IgG. **(B) **DREAM antibody immunoprecipitated DREAM, but no NMDA receptor subunits (NR1, NR2A or NR2B) was detected in the immunoprecipitation. **(C) **PSD-95 protein was immunoprecipitated from mouse hippocampal extracts by a specific DREAM antibody. Increasing Ca^2+ ^concentrations (0.1 and 0.25 mM) prevent this interaction. Absence of Ca^2+ ^(0) corresponds to 2 mM EDTA.

Since DREAM nuclear repressor activity appears not to be responsible for the decrease in NMDA receptor function, we next investigated whether DREAM could directly interact with NMDA receptor subunits or interfere in the interaction of the receptor with docking proteins known to participate in receptor functions. Among them, PSD-95 is a major scaffolding protein in the postsynaptic density, tethering NMDA receptors to signaling proteins, and is therefore critical for NMDA receptor function [49]. Co-immunoprecipitation experiments using a monoclonal antibody specific for DREAM showed that DREAM is associated with PSD-95 in hippocampal extracts from wild-type mice (Figure 6A). In parallel experiments, we did not observe the interaction between DREAM and the NMDA receptor subunits NR1, NR2A or NR2B (Figure 6B). Furthermore, the role of Ca^2+ ^in the interaction between DREAM and PSD-95 was studied. We found that the immunoprecipitation of PSD-95 was reduced when Ca^2+ ^is increased (Figure 6C). Taken together, these data indicate there is a specific interaction between DREAM and PSD-95, and Ca^2+ ^negatively regulates the interaction. The results suggest that DREAM possibly inhibits NMDA receptor function under basal conditions but allow receptor function normally upon membrane depolarization and calcium entry.

### Normal AMPA receptor function in TgDREAM mice

**Figure 7:**
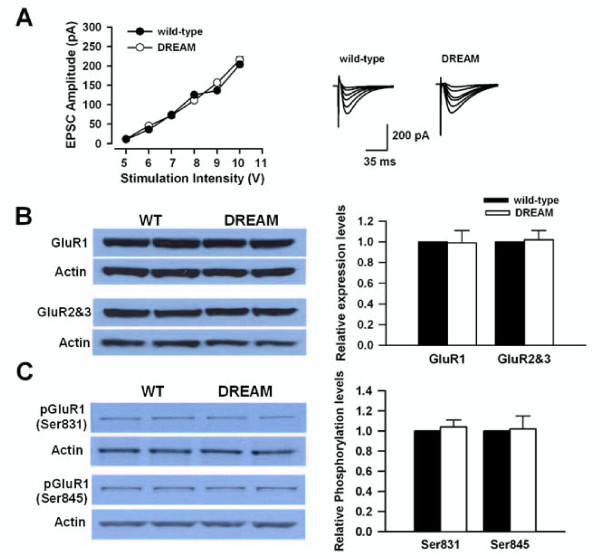
**Normal AMPA receptor function and expression inTgDREAM mice**. (A) Input-output relationship for AMPA receptor-mediated EPSCs in wild-type (filled circles, n = 7) and TgDREAM mice (open circles, n = 7). There is no significant difference between the two groups. The sample traces are shown in the right panel. (B) Representative western blot (left) and quantified data (right) for expression levels of GluR1 and GluR2&3 subunits in hippocampus from wild-type and TgDREAM mice. Data were normalized to expression level of wild-type mice (n = 4 for each group). (C) Representative western blot (left) and quantified data (right) for phosphorylation of AMPA GluR1 receptor at ser831 and 845 sites in hippocampus from wild-type and TgDREAM mice. Data were normalized to expression level of wild-type mice (n = 4 for each group).

To test whether the function of AMPA receptors was altered also in TgDREAM mice, we compared the input-output relationship of AMPA receptor-mediated EPSCs in TgDREAM and wild-type mice. No significant difference was found in AMPA EPSCs between the two genotypes (Figure 7A). In addition, we studied the expression of AMPA receptor subunits in TgDREAM mice by using Western blot. We found that the expression of GluR1 or GluR2&3 subunits was similar in TgDREAM and wild-type mice (Figure 7B). Phosphorylation of GluR1 at Ser 831 and Ser 845 sites is important for GluR1 trafficking [50]. However, there is no significant difference in pGluR1 phosphorylation at either Ser 831 or Ser 845 site in TgDREAM and wild-type mice (Figure 7C). Therefore, the loss of LTD may arise from impaired functions of NMDA receptor but not AMPA receptor and the reduced NMDA receptor function may be due to the constitutive binding of mutant DREAM with PSD-95.

### Impaired contextual fear memory in TgDREAM mice

**Figure 8:**
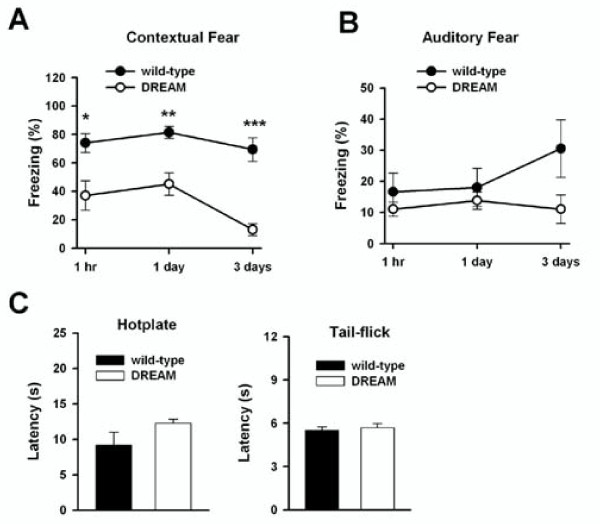
**Impaired contextual fear memory in TgDREAM mice**. **(A) **Contextual fear memory was impaired in TgDREAM mice (n = 8) compared with wild-type mice (n = 6) 1 h and 1 and 3 d after training. *** *P *< 0.001. **(B) **No significant difference in auditory fear conditioning between TgDREAM (n = 8) and wild-type mice (n = 6). **(C) **Comparison of nociceptive responses between TgDREAM (n = 8) and wild-type mice (n = 6). There was no significant difference in response latency between genotypes in the hotplate test (left) or in the tail-flick reflex (right).

It is believed that alterations in the strength of synaptic connections in hippocampus underlie contextual and spatial memories [10,51] (Table 1). Therefore, impaired NMDA functions and LTD may lead to a deficit in forebrain-dependent memory in TgDREAM mice. To test this idea, we examined contextual fear memory. We found no significant difference in freezing responses immediately following the shock/tone pairing between TgDREAM and wild-type mice, suggesting that DREAM mutation did not cause any developmental defects that would interfere with the shock-induced freezing responses. In contrast, TgDREAM mice showed a significant reduction in contextual memory at 1 hr, 1 and 3 days after conditioning compared to wild-type (n = 8 for TgDREAM; n = 6 for control) (Figure 8A). Furthermore, we tested auditory fear conditioning in TgDREAM mice and we found that auditory fear memory in transgenic mice (n = 8) is similar to that in wild-type mice (n = 6) (Figure 8B). We noticed that the freezing responses are small in both wild-type and TgDREAM mice (both are B6CBAF1/J). This may be due to the genetic variability since we have also tested C57BL/6J wild-type mice and found they show normal auditory fear responses (around 70%).

**Table 1:** Examples of genetic manipulations on hippocampal CA1 LTD and fear memory

Genetic manipulations	CA1 LTP	CA1 LTD	Basal transmission	Contextual fear memory	Auditory fear memory	References
TgDREAM	Normal	Reduced	Normal	Reduced	Normal	This study
SV40 transgenic	Normal	Reduced	Normal	Normal	-	[72]
Neurabin^-/-^	Reduced	Normal	Enhanced	Reduced	Normal	[68]
Shank1^-/-^	Normal	Normal	Reduced	Reduced	Normal	[76]
MeCP2^308/Y^	Reduced	Reduced	Enhanced	Reduced	-	[67]
EGR1^-/-^	Normal	Normal	-	Normal	Reduced	[77,78]
NCX2^-/-^	Enhanced	Lower threshold	Normal	Enhanced	Normal	[69]
CaMKIV^-/-^	Reduced	Normal	Normal	Reduced	Reduce	[23,25]
Calcineurin^-/-^	Normal	Reduced	Normal	Normal	Normal	[71]
NMDA NR2B overexpression	Enhanced	Normal	Normal	Enhanced	Enhanced	[70]
PKCγ^-/-^	Reduced	Normal	Normal	Reduced	Normal	[65,66]

To determine whether the decrease in fear memory of TgDREAM mice is attributable to changes in pain sensitivity to the footshock, we measured nociceptive responses in the hotplate and tail-flick tests. No difference was found between tail-flick latencies of wild-type and TgDREAM mice (wild-type, 5.5 ± 0.02 s, n = 6; TgDREAM, 5.7 ± 0.3 s, n = 8) (Figure 8C). Similarly, no difference was observed between nociceptive responses of wild-type and TgDREAM mice for the hotplate set at 55°C (wild-type, 9.2 ± 1.8 s, n = 6; TgDREAM, 12.3 ± 0.5 s, n = 8) (Figure 8C).

## Discussion

**Figure 9:**
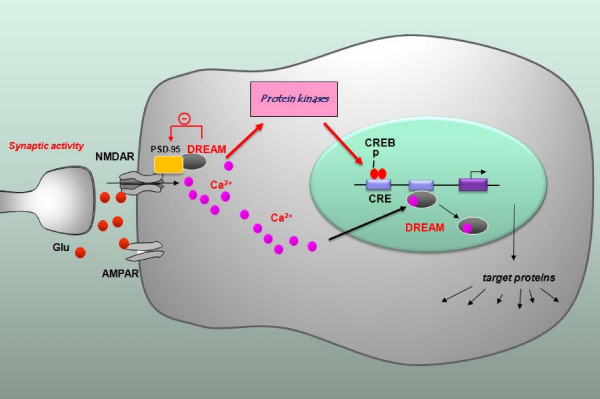
**Simplified model of the novel function of DREAM in NMDA receptor-mediated signaling transduction at synapses**. At postsynaptic sites, DREAM interacts with PSD-95, and inhibits the functions of NMDA receptors. Binding of Ca^2+ ^to DREAM releases the interaction between DREAM and PSD95. In TgDREAM mice, Ca^2+^-insensitive mutant DREAM may constitutively bind to PSD95, leading to the impaired recruitment of PSD-95 and reduced NMDA receptor function. The loss of LTD in TgDREAM mice is likely due to the impaired function of NMDA receptors. Within the nucleus, it is known that DREAM binds to DRE located downstream from the transcriptional start site, and inhibits the promoter activity. The binding of Ca^2+ ^to DREAM releases this inhibition, leading to higher levels of transcription.

DREAM was identified as the first Ca^2+^-binding protein that in the nucleus binds directly to DNA at specific regulatory elements and represses transcription in a Ca^2+^-dependent manner [34]. Outside the nucleus DREAM modifies the activity of several proteins to modulate different biological functions [32]. In the present study, we used transgenic mice overexpressing a dominant active Ca^2+^- and cAMP-insensitive mutant of DREAM to investigate the role of DREAM in synaptic transmission and plasticity in the hippocampus as well as its related learning behavioral responses. We showed that there is impaired LTD in TgDREAM mice, which may be due, at least in part, to a reduced function of NMDA receptor by the interaction between DREAM and PSD-95 (Figure 9). Finally, the contextual fear memory was significantly reduced as well. Thus, our results provide the strong evidence of DREAM involvement in regulating hippocampal NMDA receptor function, long-term plasticity and contextual fear memory.

### DREAM and hippocampal LTD

DREAM regulates the expression of several learning-related genes[32,33]. Studies using DREAM knockout mice, however, have reported no effect [35] or a minor role of DREAM in hippocampal synaptic plasticity [41]. In this later study, enhanced LTP in the dentate gyrus of DREAM knockout mice is associated with decreased A-type current density [41]. We found that LTD but not LTP was impaired in transgenic CA1 neurons. The reduced synaptic LTD in TgDREAM CA1 neurons may be due to an impaired function of NMDA receptor rather than Kv4 channel. Our conclusion is based on the following three lines of evidence: (1) Induction of synaptic plasticity at hippocampal CA1 synapses requires the activation of NMDA receptors; (2) NMDA receptor-mediated EPSCs were reduced in TgDREAM mice; (3) A-type current is not altered in TgDREAM mice. Nevertheless, additional mechanisms could also contribute to an abnormal synaptic plasticity in transgenic CA1 hippocampal neurons, e.g. the reduced expression of Na^+^/Ca^2+ ^exchanger-3 in TgDREAM hippocampus that results in elevated intracellular levels of free Ca^2+ ^[43]. Since hippocampal CA1 LTP is also NMDA receptor-dependent [21,52,53], it is puzzling that LTP is normal in TgDREAM mice. Further studies are needed to address the differential modulation of DREAM on hippocampal LTP and LTD. Moreover, different types of LTD, such as metabotropic glutamate receptor- and muscarinic acethycholine receptor-dependent LTD, have been reported in the hippocampus [54] and some of them are even Ca^2+^-independent LTD [55]. Whether or not DREAM is involved in these different forms of LTD remains to be tested in future studies. In the present study, we propose that DREAM binds with PSD95 and thus affects NMDA receptor function and the induction of LTD. In addition, there is other calcium sensing proteins, such as hippocalcin and neuronal calcium sensor-1, contribute to hippocampal LTD. Furthermore, they may attribute to hippocampal LTD by affecting the trafficking of AMPA receptor through the interaction with clathrin adaptor protein 2 complex and protein interacting with C kinase, respectively [56,57].

### DREAM and NMDA receptor functions

NMDA receptors are involved in excitatory synaptic transmission and plasticity associated with a variety of brain functions, from memory formation to chronic pain. We found that DREAM selectively regulates NMDA receptor function. Several possibilities might contribute to the regulation of NMDA EPSCs by DREAM. First, expression or phosphorylation of NMDA receptors may be altered in TgDREAM mice. This possibility could also be excluded based on our results showing no change in expression or phosphorylation of any of the NMDA receptor subunits. Second, DREAM may affect NMDA receptor function through the control of prodynorphin gene expression [34,35]. Previous studies have revealed that dynorphin could regulate the function of NMDA receptors by acting directly on the receptor [58] or indirectly through *k*-opiate receptors [59]. However, in our experimental conditions application of dynorphin did not affect NMDA-induced EPSCs in hippocampal neurons (Wu et al., unpublished data). Third, DREAM may affect NMDA receptor function by changing the expression or the function of scaffolding proteins. We found that the expression of PSD-95 is not changed in TgDREAM mice, however, co-immunoprecipitation experiments show that there is a direct interaction between DREAM and PSD-95. Binding of PSD95 with NR2 subunit was implicated in NMDA receptor trafficking and function on the cell membrane. Therefore, Ca^2+^-insensitive TgDREAM may constitutively interact with PSD-95 and inhibit NMDA receptor trafficking or there is an impaired recruitment of TgDREAM-bound PSD-95 to synaptic protein complexes needed for NMDA receptor function. Future studies are needed to explore the molecular mechanisms underlying the interaction between DREAM and PSD95 and how this interaction affects NMDA receptor function.

### Hippocampal LTD and fear memory

Since the role of the hippocampus is well established in the context-related memory formation, this experimental paradigm has been commonly used for measuring behavioral consequences of molecular changes in hippocampal circuits [10,51,60-64]. A variety of studies have shown that LTP in hippocampal CA1 neurons is correlated with contextual fear memory (Table 1). For example, reduced CA1 LTP is selectively associated with impaired contextual but not auditory fear memory in PKCγ knockout mice, CaMKIV knockout mice, MeCP2^308/Y ^transgenic mice and neurabin knockout mice [23,25,65-68], while enhanced CA1 LTP is also associated with enhanced contextual fear response in mice overexpressing NMDA NR2B or lacking Na^+^/Ca^2+ ^exchanger 2 [69,70]. In most of these cases, hippocampal LTD was not affected in these mutant mice. In mice lacking calcineurin or with expression of small t antigen of simian virus 40 inhibiting PP2A, LTD is selectively reduced. Interestingly, these mice show normal contextual fear memory but impaired working memory [71,72]. In the present study, we found that hippocampal CA1 LTD was significantly reduced in TgDREAM mice while hippocampal LTP was not affected. In parallel, behavioral contextual memory was significantly reduced, while auditory fear memory was not affected. In addition, our preliminary data using Morris water maze test also indicated the impaired spatial memory in TgDREAM mice (Mellström et al., submitted). These results provide the evidence linking hippocampal CA1 LTD with contextual fear memory. We propose that both LTP and LTD are critical for the formation of contextual fear memory, and deficits in either of them will directly or indirectly impair the memory formation.

## Materials and methods

### Animals

Transgenic mice overexpressing a Ca^2+ ^insensitive DREAM mutant (line 33) with specific dominant active function over DRE-mediated transcription have been previously described [43,44]. Wild-type and transgenic mice were housed under a 12 h light/dark cycle with food and water ad libitum. The Animal Care and Use Committee at the University of Toronto approved the experimental protocols.

### Whole-cell patch clamp recording

Coronal brain slices (300 μm) from 6- to 8-week-old C57BL/6 and TgDREAM mice containing hippocampus were prepared using standard methods [68]. Slices were transferred to a submerged recovery chamber with oxygenated (95% O_2 _and 5% CO_2_) artificial cerebrospinal fluid (ACSF) containing the following (in mM): 124 NaCl, 2.5 KCl, 2 CaCl_2_, 2 MgSO_4_, 25 NaHCO_3_, 1 NaH_2_PO4, and 10 glucose, at room temperature for at least 1 h before electrophysiological experiments. Experiments were performed in a recording chamber on the stage of an Olympus BX51WI microscope (Tokyo, Japan) with infrared DIC optics for visualization of whole-cell patch clamp recordings. Excitatory postsynaptic currents (EPSCs) were recorded from hippocampal CA1 pyramidal neurons with an Axon 200B amplifier (Molecular devices, CA) and the stimulations were delivered by a bipolar tungsten stimulating electrode placed in stratum radium. EPSCs were induced by repetitive stimulations at 0.02 Hz, and neurons were voltage clamped at -70 mV. Picrotoxin (100 μM) was always present to block GABA_A _receptor-mediated inhibitory synaptic currents. The recording pipettes (3-5 MΩ) were filled with solution containing (in mM):145 K-gluconate, 5 NaCl, 1 MgCl_2_, 0.2 EGTA, 10 HEPES, 2 Mg-ATP, and 0.1 Na_3_-GTP (adjusted to pH 7.2 with KOH). When current-voltage relationship experiments and NMDA receptor-mediated EPSCs were recorded, K-gluconate was replaced by equomolar CsMeSO_3 _and 5 mM QX-314 chloride in the pipette solution. The NMDA EPSCs were recorded at the holding potential of -10 mV in the presence of CNQX (20 μM). NMDA EPSCs were evoked at 0.05 Hz. After obtaining stable EPSCs for at least 10 min, LTP was induce by 300 pulses at 2 Hz paired with postsynaptic depolarization at +30 mV, while LTD was induced by 300 pulses at 1 Hz paired with postsynaptic depolarization at -45 mV [73,74]. Throughout the experiment, the series resistance was continuously monitored and data were discarded if series resistance changed by more than 15% during experiments.

### Western blot

Hippocampal tissues were dissected and homogenized in lysis buffer (10 mM Tris-HCl, pH 7.4, 2 mM EDTA, 1% SDS) containing 1× protease inhibitor cocktail (Sigma, MO) and 1× phosphatase inhibitor cocktail 1 and 2 (Sigma, MO). Western blot analysis was carried out as described [75]. Briefly, protein samples were separated on SDS-polyacrylamide gels and transferred to polyvinylidene fluoride membranes at 4°C. Membranes were probed with 1:3000 dilution of anti-GluR1 (Upstate, NY), anti-GluR2&3 (Chemicon, CA), 1:2000 dilution of anti-PSD-95 (Chemicon, CA), 1:1000 dilution of anti-phospho-GluR1 Ser845 (Upstate, NY) or anti-phospho-GluR1 Ser831 (Upstate, NY), and 1:1000 dilution of anti-NR1 (Upstate, NY) anti-NR2A (Chemicon), or anti-NR2B (Chemicon) antibodies. The membranes were incubated in the appropriate horseradish peroxidase-coupled secondary antibody for 1 h followed by enhanced chemiluminescence (ECL) detection of the proteins with Western Lightning Chemiluminescence Reagent Plus (Perkin Elmer Life Sciences, MA) according to the manufacturer's instructions. To verify equal loading, membranes were also probed with 1:3000 dilution of anti-actin antibody (Sigma, MO). The density of immunoblots was measured using the NIH ImageJ software.

### Quantitative real-time RT-PCR

RNA was isolated from hippocampal tissues using TRIzol (Invitrogene), treated with DNAse (Ambion) and reverse transcribed using hexamer primers and Moloney murine leukemia virus reverse transcriptase (Invitrogene). To confirm the absence of genomic DNA, each sample was processed in parallel without reverse transcriptase. Quantitative real-time PCR for TgDREAM was performed using the primers: forward 5'-CACCTATGCACACTTCCTCTTCA-3' and reverse 5'-ACCACAAAGTCCTCAAAGTGGAT-3' and the TaqMan MGB probe VIC-5'-CGCCTTTGCTGCGGC-3'-MGB, specific for TgDREAM. The results were normalized by quantification of β-actin mRNA using specific primers and TaqMan MGB probe supplied by Applied-Biosystems.

### Immunohistochemistry

Animals (n = 6) were overdosed with sodium pentobarbital and perfused transcardially with 20 ml of 0.1 M phosphate buffered saline (PBS; pH = 7.4) and 4% paraformaldehyde in PBS. Brains were then dissected out, cryoprotected in 30% sucrose, included in embedding medium (Tissue-Tek; Sakura Finetek, Torrance, CA), fast-frozen in dry-ice, cut coronally on a cryostat (20 μm), thaw-mounted on glass slides and allowed to dry overnight. Sections were re-hydrated by incubation in alcohol solutions of decreasing concentrations (100, 95, 70, 50%; 2 mins each) and placed in distilled water for 5 mins. Next, sections were placed in a 0.5% Cresyl violet solution for 5 mins, dehydrated in a series of alcohols, defatted in xylenes and coverslipped.

### Immunoprecipitation

To detect NMDA receptor subunit phosphorylation, solubilized protein samples were prepared with modified RIPA buffer (50 mM Tris-HCl, pH 7.4, 1% NP-40, 0.25% Na-deoxycholate, 150 mM NaCl, 1 mM EDTA, 1 mM PMSF), and precipitated with 50 μl of protein G-agarose pre-coupled with anti-phosphoserine (BD Biosciences, Franklin Lakes, NJ) for 4 h at 4°C. The reaction mixtures were then washed three times and eluted by boiling in loading buffer, and were subjected to western blot using the antibodies to NMDA receptor subunits.

The interaction between DREAM and PSD-95 was shown by coimmunoprecipitation from hippocampal tissue, homogenized and lysed by in buffer: Tris pH 7.5, 50 mM; NaCl 150 mM; NP40 1%; Na-deoxycholate 0.25%; protease inhibitor cocktail. Cleared extracts were supplemented with EDTA, 2 mM or with CaCl_2_, 0.1 and 0.25 mM final concentrations. Five μg of a specific antibody for DREAM was used for immunoprecipitation and immuncomplexes were captured using protein A-coupled magnetic beads (Ademtech). Samples without specific antibody were used as control washed beads were eluted and subjected to western blot using anti-PSD-95 (clone 7E3-1B8, Affinity BioReagents) 1/2000.

### Fear conditioning

Fear conditioning was performed in an isolated shock chamber (Med Associates, St. Albans, VT). Freezing responses (total immobility aside from respiration) were manually scored every 10 s. The conditioned stimulus (CS) was an 85 dB sound at 2,800 Hz for 30 s, and the unconditioned stimulus (US) was a continuous scrambled foot shock at 0.75 mA for 2 s. During training, after 2 min of habituation, mice were presented with a 30 s tone (CS) and a shock (US) beginning at 28 s after the onset of CS. After CS/US pairing (three tone-shock pairings were delivered at 30 s intervals), the mice remained in the chamber for an additional 30 s to measure immediate freezing. At 1 h, 1 and 3 d after training, each mouse was placed back into the shock chamber to test retention, and the freezing response was recorded for 3 min (contextual conditioning). Subsequently, the mice were placed in a novel chamber and monitored for 3 min before the onset of the tone identical to the CS was delivered for 3 min, and the freezing response was recorded (auditory conditioning).

### Behavioral sensory responses to noxious stimuli

The spinal nociceptive tail-flick reflex was evoked by focused, radiant heat (Columbus Instruments, Columbus, Ohio) provided by a 50 W projector lamp focused on a 1.5 × 10 mm area on the underside of the tail. The cutoff time of 10 seconds was used to minimize damage to the skin of the tail. The hotplate consisted of a thermally controlled 10-inch × 10-inch metal plate (55°C) surrounded by four Plexiglass walls (Columbus Instruments, Columbus, Ohio). The cutoff time of 30 seconds was imposed to prevent tissue damage. All behavioral tests were performed at 10 min intervals. The baseline response latency was an average of three or four measurements.

### Data analysis

Results were expressed as mean ± SEM. Statistical comparisons were performed using one- or two-way ANOVA using the Student-Newman-Keuls test for post hoc comparisons.

## Conflicts of interests

The authors declare that they have no competing interests.

## Authors' contributions

LJW, MR and XYL carried out electrophysiological and imaging experiments, BM, HW and SD performed biochemical experiments, TC performed staining experiments, SSK performed behavioral experiments, LJW and MZ drafted the manuscript and coordinated the study. All authors read and approved the final manuscript.
